# Acetazolamide-mediated decrease in strong ion difference accounts for the correction of metabolic alkalosis in critically ill patients

**DOI:** 10.1186/cc3970

**Published:** 2006-01-09

**Authors:** Miriam Moviat, Peter Pickkers, Peter HJ van der Voort, Johannes G van der Hoeven

**Affiliations:** 1 Department of Intensive Care Medicine, Radboud University Nijmegen Medical Centre, Nijmegen, The Netherlands; 2 Department of Intensive Care Medicine, Medical Centre Leeuwarden

## Abstract

**Introduction:**

Metabolic alkalosis is a commonly encountered acid–base derangement in the intensive care unit. Treatment with the carbonic anhydrase inhibitor acetazolamide is indicated in selected cases. According to the quantitative approach described by Stewart, correction of serum pH due to carbonic anhydrase inhibition in the proximal tubule cannot be explained by excretion of bicarbonate. Using the Stewart approach, we studied the mechanism of action of acetazolamide in critically ill patients with a metabolic alkalosis.

**Methods:**

Fifteen consecutive intensive care unit patients with metabolic alkalosis (pH ≥ 7.48 and HCO_3_^- ^≥ 28 mmol/l) were treated with a single administration of 500 mg acetazolamide intravenously. Serum levels of strong ions, creatinine, lactate, weak acids, pH and partial carbon dioxide tension were measured at 0, 12, 24, 48 and 72 hours. The main strong ions in urine and pH were measured at 0, 3, 6, 12, 24, 48 and 72 hours. Strong ion difference (SID), strong ion gap, sodium–chloride effect, and the urinary SID were calculated. Data (mean ± standard error were analyzed by comparing baseline variables and time dependent changes by one way analysis of variance for repeated measures.

**Results:**

After a single administration of acetazolamide, correction of serum pH (from 7.49 ± 0.01 to 7.46 ± 0.01; *P *= 0.001) was maximal at 24 hours and sustained during the period of observation. The parallel decrease in partial carbon dioxide tension was not significant (from 5.7 ± 0.2 to 5.3 ± 0.2 kPa; *P *= 0.08) and there was no significant change in total concentration of weak acids. Serum SID decreased significantly (from 41.5 ± 1.3 to 38.0 ± 1.0 mEq/l; *P *= 0.03) due to an increase in serum chloride (from 105 ± 1.2 to 110 ± 1.2 mmol/l; *P *< 0.0001). The decrease in serum SID was explained by a significant increase in the urinary excretion of sodium without chloride during the first 24 hours (increase in urinary SID: from 48.4 ± 15.1 to 85.3 ± 7.7; *P *= 0.02).

**Conclusion:**

A single dose of acetazolamide effectively corrects metabolic alkalosis in critically ill patients by decreasing the serum SID. This effect is completely explained by the increased renal excretion ratio of sodium to chloride, resulting in an increase in serum chloride.

## Introduction

Metabolic alkalosis is a common acid–base disturbance in the intensive care unit (ICU) that is associated with increased ICU mortality and morbidity [1,2], with adverse effects on cardiovascular, pulmonary and metabolic function [3,4]. Additionally, such patients are characterized by compensatory alveolar hypoventilation, which can result in delayed weaning from mechanical ventilation. Options for treatment aimed at correcting metabolic alkalosis are fluid and potassium replacement, and administration of ammonium chloride, hydrochloric acid, or acetazolamide [5]. These therapeutic interventions potentially increase minute ventilation, allowing patients to be weaned more rapidly [6].

An advanced understanding of acid–base physiology is central to the practice of critical care medicine. Although it is not difficult to quantify the degree of metabolic alkalosis, it is more challenging to identify the cause of a metabolic alkalosis and determine the actions that must be taken to correct it. The method of quantifying and qualifying an acid–base disturbance, as described by Stewart, relies on the accepted physicochemical principles of conservation of mass and electroneutrality [7,8]. According to Stewart, three variables independently determine the serum hydrogen concentration. These variables are the partial carbon dioxide tension (PCO_2_), the total concentration of nonvolatile weak acids (primarily serum proteins and phosphate), and the strong ion difference (SID) [9]. The Stewart approach, in contrast to other approaches, allows us to quantify an acid–base derangement as well as determine its cause.

The kidneys are the most important regulators of SID for acid–base purposes. The concentration of strong ions in plasma can be altered by adjusting absorption from glomerular filtrate or secretion into the tubular lumen from plasma. In this respect, administration of the carbonic anhydrase inhibitor acetazolamide during metabolic alkalosis could modulate plasma pH by influencing the urinary excretion of various strong ions. Because plasma sodium controls intravascular volume and osmolality, and because plasma potassium is important for cardiac and neuromuscular function, plasma chloride appears to represent the strong ion that the kidney uses to regulate acid–base status without interfering with other important homeostatic processes [7]. Furthermore, the basic physicochemical principles imply that a change in bicarbonate concentration is not a cause but merely a co-phenomenon of an acid–base disturbance such as metabolic alkalosis.

Acetazolamide decreases proximal tubular bicarbonate reabsorption by up to 80% through inhibition of carbonic anhydrase in the luminal borders of renal proximal tubule cells, and it is often effectively used in the treatment of metabolic alkalosis in the ICU. However, the mechanism of action of acetazolamide remains unclear. According to the basic physicochemical principles mentioned above, retention of bicarbonate cannot causally be related to correction of serum pH, and acetazolamide-induced effects must be explained by modulation of the urinary excretion of strong ions.

We hypothesized that acetazolamide, by inhibiting carbonic anhydrase in the proximal tubules, causes excretion of strong cations (along with bicarbonate) and retention of chloride, and in this way decreases the serum SID. Subsequently, the decrease in SID will correct an alkalosis by causing dissociation of water and formation of hydrogen ions. The purpose of the present study was to determine the mechanism of action of acetazolamide in critically ill patients with a metabolic alkalosis according to the physicochemical principles described by Stewart.

## Materials and methods

### Patients

The local ethics committee granted approval for the study and, because the indication for acetazolamide was based on clinical grounds, waived the need for informed consent. This prospective study was set in the multidisciplinary ICU of the Radboud University Nijmegen Medical Centre, Nijmegen, The Netherlands.

We studied 15 consecutive ICU patients with a metabolic alkalosis (defined as pH ≥ 7.48) and serum bicarbonate of 28 mmol/l or greater. All patients had an arterial line *in situ*. Patients clinically suspected of having volume contraction (for example, cold extremities, blood pressure increase during passive leg raising), hypokalaemia (serum potassium ≤ 3.4 mmol/l), nasogastric tube drainage greater than 50 cc/hour, renal insufficiency (creatinine clearance <20 ml/min and/or renal replacement therapy), or intolerance or allergy to acetazolamide or sulfonamides were excluded. Also excluded were patients who were treated with intravenous acetazolamide or sodium bicarbonate during the previous 72 hours.

Acute Physiology and Chronic Health Evaluation II score was calculated and recorded for each patient for the first 24 hours after admission. Data on fluid intake and output, ventilator settings, and relevant medications such as diuretics and steroids were also recorded. After inclusion, patients received a single dose acetazolamide (500 mg as an intravenous push).

### Experimental design

We measured pH, arterial oxygen tension, arterial PCO_2_, sodium, potassium, chloride, magnesium, calcium, lactate, creatinine, urea, phosphate and albumin in a single arterial blood sample before acetazolamide was administered (t = 0) and 12, 24, 48 and 72 hours later (t = 12, t = 24, t = 48 and t = 72).

Urine samples were taken before acetazolamide was administered, and 3, 6, 12, 24, 48 and 72 hours later. In these samples pH was measured immediately. Urine was stored at -80°C, and sodium, chloride, potassium and creatinine were measured in a single batch at the end of the study.

### Data analysis, calculations and statistics

Bicarbonate was calculated using the Henderson–Hasselbalch equation (pH = 6.1 + log [HCO_3_^-^]/0.0301 arterial PCO_2_]) and the standard base excess was calculated using the Siggaard–Andersen formula.

The apparent SID (SID_app_) was calculated using the equation SID_app _= [Na^+^] + [K^+^] + [Ca^2+^] + [Mg^2+^] - [Cl^-^] - [lactate^-^]. The effective SID (SID_eff_) was calculated using the equation SID_eff _= 12.2 × PCO_2_/(10^-pH^) + [albumin] × (0.123 × pH - 0.631) + [PO_4_^-^] × (0.309 × pH - 0.469). The strong ion gap (SIG) was calculated using the equation SIG = SID_app _- SID_eff _[10]. The sodium–chloride effect was calculated using the formula [Na^+^] - [Cl^-^] - 38 [11]. Urinary electrolytes to creatinine ratios and sodium to chloride ratios were calculated, as was the urinary SID using the following equation: urinary SID = [Na^+^] + [K^+^] - [Cl^-^].

The effects of acetazolamide were analyzed by comparing baseline variables and time-dependent changes using one-way analysis of variance with repeated measures. Power analysis was based on a presumed standard deviation of 15% for the measured end-points. A change of 10% was considered clinically relevant. With α = 0.05, we calculated that a sample size of 14 would be needed to achieve a power of 80%. Therefore, 15 patients were included.

Data are expressed as mean ± standard error unless otherwise specified. *P *< 0.05 was considered statistically significant.

## Results

### Patients

Patient characteristics are presented in Table 1, and baseline acid–base and electrolyte data are presented in Table 2. Of the patients studied, 87% were mechanically ventilated (all in an assisted mode of ventilation in which spontaneous breathing activity was fully possible). Although 47% of the patients were treated with diuretics, none exhibited clinical symptoms of hypovolaemia. Furthermore, low urinary chloride excretion (< 20 mmol/l), which is indicative of hypovolaemia in patients who do not use diuretics, was present in only one patient. Intravenous and enteral intake of sodium chloride, as well as ventilator settings and diuretic dose, were not changed during the study period.

### Effects of acetazolamide on Stewart's parameters in blood

**Figure 1:**
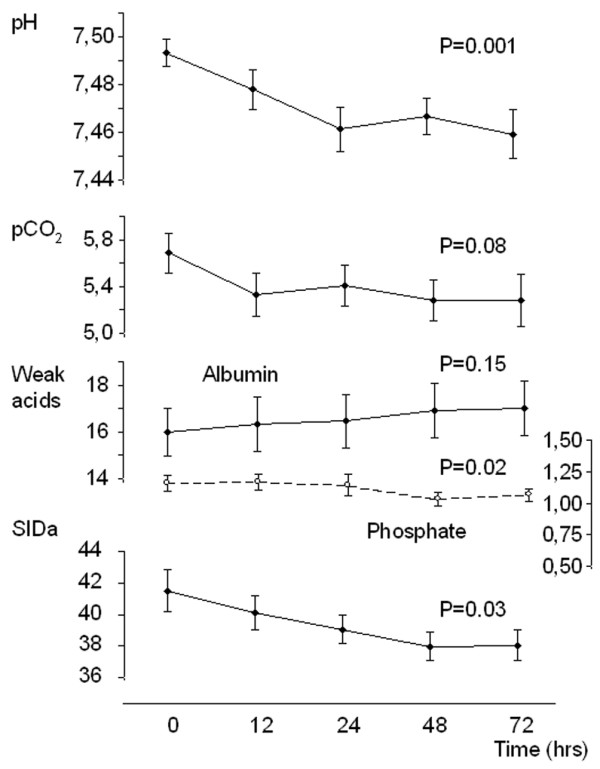
Time course of acetazolamide-induced changes in pH and three independent variables that determine pH. Effect of 500 mg acetazolamide administration (intravenous) in patients with metabolic alkalosis. Data are expressed as mean ± standard error values for 15 patients. The *P *values refer to the time-dependent changes analyzed using one-way analysis of variance. pCO_2_, partial carbon dioxide tension; SIDa, apparent strong ion difference.

After administration of acetazolamide, correction of serum pH (7.49 ± 0.01 to 7.46 ± 0.01; *P *= 0.001) was maximal at 24 hours and was sustained during the period of observation (Figure 1). The parallel decrease in PCO_2 _was not significant (from 5.7 ± 0.2 to 5.3 ± 0.2 kPa; *P *= 0.08). There was no significant change in the total concentration of weak acids. When values of weak acids were expressed as values contributing to the electrical charge equilibrium in plasma (see formula for SID_eff_), phosphate decreased from 2.14 ± 0.11 mEq/l to 1.94 ± 0.10 mEq/L (*P *= 0.02), and albumin remained unchanged (from 4.65 ± 0.30 mEq/l to 4.87 ± 0.35 mEq/L; *P *= 0.15; Figure 1).

**Figure 2:**
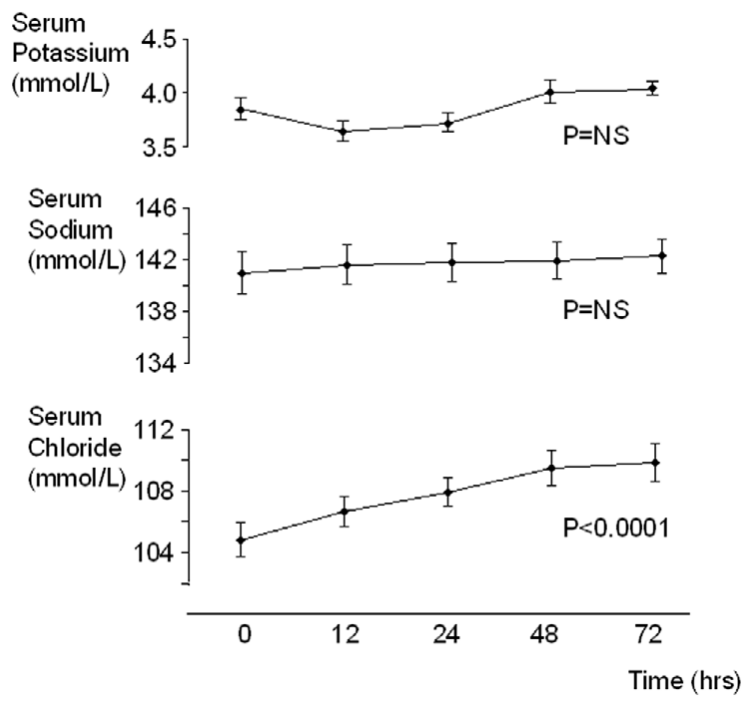
Time course of acetazolamide-induced changes in serum potassium, sodium and chloride. Effect of 500 mg acetazolamide administration (intravenous) in patients with metabolic alkalosis. Serum chloride exhibited a significant increase, whereas there were no significant changes in serum potassium and sodium concentration. Data are expressed as mean ± standard error values for 15 patients. The *P *values refer to the time-dependent changes analyzed using one-way analysis of variance.

Serum SID decreased significantly during the period of observation (from 41.5 ± 1.3 mEq/l to 38.0 ± 1.0 mEq/l; *P *= 0.03) because of an increase in serum chloride (from 105 ± 1.2 mmol/l to 110 ± 1.2 mmol/l; *P *< 0.0001, figure 2). There was a strong relation between the serum SID and the sodium–chloride effect (R^2 ^= 0.99; *P *< 0.001), indicating that the observed changes in SID are completely accounted for by changes in serum sodium and/or chloride and not other strong ions. The decrease in serum SID was caused by a significant increase in the urinary excretion of sodium without chloride during the first 24 hours (change in urinary [Na]/[Cl]: from 1.3 ± 0.3 to 2.5 ± 0.5; *P *= 0.02), resulting in an increase in urinary SID (see Effects of acetazolamide on Stewart's parameters in urine, below).

In the patients studied here, there was no relevant SIG (mean baseline value 2.11 ± 0.81 mEq/L), and it exhibited no change after administration of acetazolamide (3.13 ± 0.48; *P *= 0.43).

### Effects of acetazolamide on Stewart's parameters in urine

**Figure 3:**
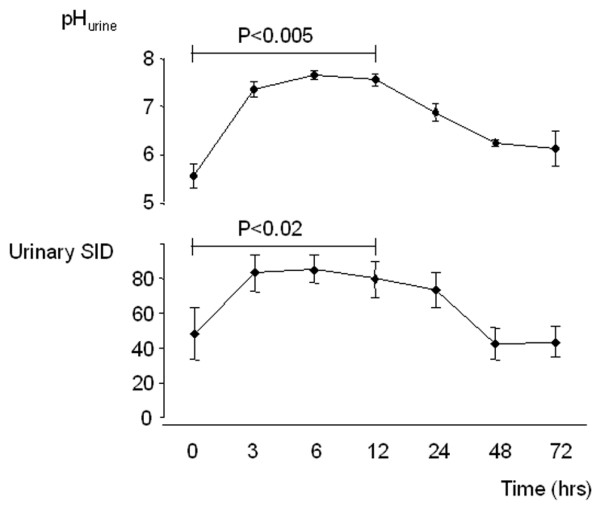
Effect acetazolamide on urinary pH and sodium–chloride ratio. Effect of 500 mg acetazolamide administration (intravenous) in patients with metabolic alkalosis. Data are expressed as mean ± standard error values for 15 patients. The *P *values refer to the time-dependent changes analyzed using one-way analysis of variance.

Urinary pH increased significantly from 5.55 ± 0.26 to 6.13 ± 0.37 (*P *= 0.005) during the first 12 hours after administration of acetazolamide, and returned to pre-administration value during the next 60 hours (Figure 3). Urinary SID exhibited a parallel increase (from 48.4 ± 15.1 to 85.3 ± 7.7; *P *= 0.02) during the first 12 hours and a parallel decrease thereafter.

## Discussion

Our study is the first to demonstrate that the acetazolamide-induced correction of metabolic alkalosis in critically ill patients can completely be accounted for by a significant decrease in serum SID, using the physicochemical principles described by Stewart. Although analysis using the Henderson–Hasselbalch equation is useful for describing and classifying acid–base disorders, the physicochemical approach described by Stewart is better suited to quantifying these disorders and for generating hypotheses regarding mechanisms.

Use of the Stewart model has improved our understanding of the pathophysiology that leads to changes in acid–base balance. SID, total concentration of nonvolatile weak acids, and PCO_2 _are biological variables that are regulated mainly by renal tubular transport, metabolism and ventilation. The relative complexity of the Stewart approach comes from the fact that several variables are needed. However, when these variables are absent or assumed to be normal, the approach becomes essentially indistinguishable from the more traditional descriptive methods. For example, our study does not dispute the contention that acetazolamide, through inhibition of carbonic anhydrase in the proximal tubule, increases urinary bicarbonate excretion. However, according to the Stewart approach it is not the loss of bicarbonate that determines the fall in pH, because bicarbonate is not an independent parameter. According to Stewart, it is the change in SID (due to a rise in chloride) that explains the decrease in pH. In our patients, acetazolamide-induced loss of bicarbonate facilitated the renal reabsorption of chloride, while sodium could still be excreted. In other words, acetazolamide-induced bicarbonate excretion permits urinary excretion of sodium without loss of any strong anions, resulting in a lower SID and thereby a decrease in pH.

Apart from the acetazolamide-induced change in SID, our study demonstrates that inhibition of carbonic anhydrase does not significantly alter the other independent determinants of serum pH. In contrast, the nonsignificant decrease in PCO_2 _and small decrease in weak acid phosphate cause the opposite effect on serum pH. The small decrease in PCO_2 _in our patients can be explained by an increase in minute ventilation in response to correction of serum pH by acetazolamide. This increase in minute ventilation, as a result of an increased respiratory drive, was possible in an assisted mode of mechanical ventilation. Finally, the observed small increase in serum albumin does not have a significant lowering effect on serum pH and could probably be explained by the hemo-concentrating effect of diuretics during the study period.

The acetazolamide-induced decrease in SID is entirely caused by a change in serum concentration of chloride, as shown by the strong relation between the SID and the sodium–chloride effect. These changes in sodium and chloride are explained by an increase in urinary sodium excretion (along with a weak anion) while chloride excretion is maintained, as shown by the increased urinary sodium–chloride ratios. The intravenous and enteral salt intake of patients was unchanged during the observation period. Thus, the renal effect of acetazolamide results in a relative increase in serum chloride. Because sodium and chloride are the most abundant and therefore the most important strong ions, an increase in chloride relative to sodium will have a significant lowering effect on serum SID. Stewart proposed that H^+ ^and therefore pH cannot change unless one or more of the three independent variables (PCO_2_, weak acids, and SID) change. Our study demonstrates that the acetazolamide-induced effects on pH are solely mediated by a decrease in serum SID through renal excretion of sodium without chloride. Although the Stewart approach has proved to be valuable in critically ill acidotic patients [12-14], this paper represents the first report using the Stewart approach during metabolic alkalosis.

Our study confirms previous reports in patients with metabolic alkalosis that, despite corrected fluid and electrolyte abnormalities, a single dose of acetazolamide is an effective and safe form of therapy, with a quick onset and long duration of action [5,15]. Our findings suggest that the duration of the pharmacologic effect of a single administration of 500 mg acetazolamide exceeds its serum half-life (6–8 hours). This long effect is reflected by the 24-hour duration of altered urinary sodium and chloride excretion. Furthermore, after normalization of serum pH at 24 hours, this correction was sustained although urinary electrolyte excretion and pH returned to pre-administration values. Apparently, once the serum SID is corrected by acetazolamide because of the increased sodium excretion without a strong anion, this new equilibrium is maintained. The Stewart approach does not help us to explain the long-lasting effects of acetazolamide, and it is unclear how the new equilibrium is maintained after correction of the SID and what the regulating mechanism is that induces the permanent hyperchloraemia. The pharmacokinetics of acetazolamide in tissue (not plasma) may explain this observation. Another explanation could be that the alkalizing factors that were originally present in our patients are corrected during the course of the observation period. Although clinical suspicion of a hypovolaemic state was an exclusion criterion in our study, one of the alkalizing factors could very well be some degree of volume contraction induced by the administration of diuretics. Whatever the cause, it is highly unlikely that the presence of some degree of hypovolaemia in our patients would influence our conclusions regarding the effects – as determined using the Stewart approach – of acetazolamide on metabolic alkalosis.

The SIG – indicative of the presence of unmeasured anions, which are often present in metabolic acidosis, particularly in patients with renal failure [14] – was not found to be elevated in our study, as was expected. Furthermore, administration of acetazolamide had no influence on the SIG.

## Conclusion

Our study is the first to report the mechanism by which acetazolamide-induced correction of metabolic alkalosis in critically ill patients is mediated. Applying the quantitative biophysical principles of acid–base analysis described by Stewart, the acetazolamide-induced effects on serum pH are completely accounted for by an increased renal excretion of sodium without chloride, resulting in an increase in serum chloride and a decrease in serum SID.

## Key messages

• 	The Stewart approach offers an advanced understanding of acid–base physiology that is central to the practice of critical care medicine.

• 	Although the Stewart approach has proved to be valuable in critically ill acidotic patients, no reports exist in which the approach is used in ICU patients with metabolic alkalosis.

• 	In ICU patients with metabolic alkalosis, the carbonic anhydrase inhibitor acetazolamide corrects pH by decreasing the SID, with no effect on the other independent determinants of pH.

• 	The decrease in serum SID is completely explained by an increase in plasma chloride, caused by an increase in the urinary excretion of sodium without chloride.

• 	The Stewart approach explains that acetazolamide-induced loss of bicarbonate is not the cause of the decrease in serum pH, but only facilitates the renal reabsorption of chloride while sodium can still be excreted.

## Abbreviations

ICU = intensive care unit; PCO_2 _= partial carbon dioxide tension; SID = strong ion difference; SIG = strong ion gap.

## Competing interests

The authors declare that they have no competing interests.

## Authors' contributions

MM collected all of the data and drafted the manuscript. PP conceived the study and cowrote the manuscript. PvdV and JGvdH participated in the design of the study and corrected the manuscript. All authors read and approved the final manuscript.

## Figures and Tables

**Table 1:** Patients characteristics

Characteristic	Value
Age (years; mean [range])	67 (35–79)
Sex (male/female; *n*)	10/5
APACHE II score (mean [range])	21 (12–30)
Mechanical ventilation (%)	87
Diuretics (%)	47
Standardized mortality ratio	0.57
Hospital mortality (%)	20
Diagnosis	
Dissecting/ruptured aorta	2
Postoperative bleeding	2
Sepsis	4
Open heart surgery	4
Cardiogenic shock	1
Neurological disease	2

Shown are demographic data of all patients. APACHE, Acute Physiology and Chronic Health Evaluation.

**Table 2:** Acid–base and electrolyte data

Acid–base and electrolyte data	Baseline	t = 24
PH	7.49 (7.48–7.51)	7.46 (7.44–7.48)
PaCO_2 _(kPa)	5.7 (5.1–6.1)	5.3 (4.9–5.9)
Bicarbonate (mmol/l)	31.5 (29.5–33.7)	28.6 (26.3–30.3)
Sodium (mmol/l)	141 (139–145)	142 (139–145)
Potassium (mmol/l)	3.7 (3.7–4)	3.8 (3.4–3.9)
Chloride (mmol/l)	106 (102–107)	108 (107–110)
Creatinine (μmol/l)	64 (49–95)	65 (49–101)
Lactate (mmol/l)	1.4 (1.2–1.8)	1.5 (1.2–1.7)
Albumin (g/l)	16 (14–20)	17 (15–20)
Apparent SID (mEq/l)	41.7 (39.1–44.0)	39.4 (36.4–41.4)
Effective SID (mEq/l)	39.0 (37.3–40.3)	35.6 (32.9–37.7)
SIG (mEq/l)	2.4 (1.5–4.4)	3.1 (2.1–4.8)
Sodium–chloride effect (mEq/l)	-2.0 (-3.5 to +0.5)	-3.0 (-7.5 to -1.5)

Shown are baseline acid–base and electrolyte data (median [interquartile range]) for 15 patients before administration of 500 mg acetazolamide (baseline) and after 24 hours (t = 24). The serum apparent SID (SID_app_) was calculated using the following equation: SID_app _= [Na^+^] + [K^+^] + [Ca^2+^] + [Mg^2+^] - [Cl^-^] - [lactate^-^]. The serum effective SID (SID_eff_) was calculated using the following equation: SID_eff _= 12.2 × PCO_2_/(10^-pH^) + [albumin] × (0.123 × pH - 0.631) + [PO_4_^-^] × (0.309 × pH - 0.469). The SIG was calculated using the following equation: SIG = SID_app _- SID_eff_. The sodium–chloride effect was calculated using the formula [Na^+^] - [Cl^-^] - 38. PaCO_2_, arterial carbon dioxide tension; SID, strong ion difference; SIG, strong ion gap.
